# Prostate cancer, insulin, and androgen deprivation therapy

**DOI:** 10.1038/sj.bjc.6601351

**Published:** 2003-10-28

**Authors:** P Stattin, R Kaaks

**Affiliations:** 1 Department of Urology and Andrology, Umeå University Hospital, S-901 85 Umeå, Sweden; 2 International Agency for Research on Cancer, Lyon, France

**Sir**,

In a recent paper, Lehrer and co-authors report that levels of serum insulin were significantly higher in men that the authors defined as high risk prostate cancer patients in comparison to patients with low or intermediate risk (Lehrer *et al*, 2002). Patients were assigned to risk category according to an evaluation of local tumour stage, tumour differentiation and serum level of prostate specific antigen (PSA). In essence, low risk patients had small tumours of high differentiation and low PSA, whereas high-risk patients had large tumours of poor differentiation and high PSA. The authors conclude that their data were suggestive of an association between insulin levels and advanced prostate cancer stage and that insulin may possibly be used as a biomarker of advanced stage in prostate cancer. Stimulated by Lehrer *et al*. we reanalysed data from a previously published study in we found no significant association between prostate cancer risk and insulin levels in blood samples obtained median time 3.9 years before diagnosis (Stattin *et al*, 2000). In contrast to what Lehrer and colleagues observed in their study, we found only mild, non-significant increases in insulin levels for the tumours with the most adverse characteristics when analysing insulin levels according to local tumour stage, tumour grade, and PSA levels (Table 1

**Table 1:** 

	**Local tumour stage^a^**				**Tumour grade^b^**				**PSA level (ng/ml)^c^**			
	**T1a-c**	**T2**	**T3, T4**	***P*diff *P*linearity**	**1**	**2**	**3**	***P*diff *P*linearity**	**<10**	**10–15**	**>15**	***P*diff *P*linearity**
*N* (%)	63 (43%)	66 (45%)	19 (14%)		76 (51%)	52 (35%)	19 (13%)		63 (43%)	29 (20%)	55 (37%)	
Insulin (SD) (*μ*Units/mL)	5.7 (13)	6.2 (7.8)	7.2 (16)	0.77	5.4 (12.3)	6.6 (12.0)	7.3 (4.4)	0.77	5.2 (4.9)	7.6 (22.6)	6.6 (6.2)	0.05
				0.50				0.52				0.33

Geometric mean of serum insulin levels and (standard deviation) in blood samples from 149 men obtained median time 3.9 years before the diagnosis with prostate cancer in a previous study (Stattin *et al*, 2000).

a Local tumour stage according to the 1992 classification of the Union International contre le cancer (UICC).

b Tumour grade according to the classification of the World Health organization (WHO).

c Serum levels of prostate specific antigen (PSA) at the time of diagnosis. Missing data for one man for tumour stage and for two men for PSA and tumour grade.

). We suggest the following interpretation of the dissimilarity: In the study by Lehrer *et al*., the sampling of blood used for measurements of serum levels of insulin was performed *after* therapy for prostate cancer had been completed, which was radioactive implant only for men with low risk, radioactive implant plus androgen deprivation therapy (ADT) three months before radiation for men with intermediate risk, and radioactive implant, external beam therapy, plus (ADT) for men with high risk. Therefore, it cannot be ruled out that the different forms of therapy given to each risk group (outcome) may have influenced the measurement of insulin (exposure). For two of the three groups therapy included ADT. There are some data in the literature on the effects of ADT and androgen substitution on insulin levels and insulin sensitivity in men. In several cross-sectional studies, serum levels of androgens have been inversely related to insulin levels and insulin sensitivity (Haffner *et al*, 1994; Thomas *et al*, 2000; Jansson *et al*, 2002). Furthermore, a recent study showed an increase in serum levels of insulin and leptin in men who underwent ADT (Nowicki *et al*, 2001), whereas in a small clinical case series, diabetes control deteriorated after ADT (Fukui *et al*, 2000). On the other hand, several studies have shown improvement in insulin sensitivity after implementation of androgen supplementation in physiological doses (Marin *et al*, 1992; Rizza, 2000). Thus, available data seem to consistently point to an inverse relationship between serum levels of androgens and insulin. Given this relationship, we propose that the increased insulin levels in the high risk group in the study by Lehrer *et al*. may, at least partially be due to the low levels of androgens in these men caused by ADT. Thus, the causality between exposure and outcome may in fact have been reversed in that study, i.e. the risk group (outcome) determined the therapy that in turn may have influenced the serum level of insulin (exposure).

However, the hypothesis that insulin may stimulate prostate cancer development and growth remains of interest, and a recent well-designed case control study in China reported a statistically significant increase in risk of prostate cancer for men with high levels of insulin (Hsing *et al*, 2001). Clearly, there is a need for further well-designed studies addressing this issue.
